# Supplementary material for: Synthesis, Characterization, Catalytic Activity, and DFT Calculations of Zn(II) Hydrazone Complexes
Source: Molecules. 2020 Sep 4;25(18):4043. doi: 10.3390/molecules25184043 (PMC7570652; doi:10.3390/molecules25184043)

checkCIF/PLATON report

Structure factors have been supplied for datablock(s) 1, 2

THIS REPORT IS FOR GUIDANCE ONLY. IF USED AS PART OF A REVIEW PROCEDURE FOR PUBLICATION, IT SHOULD NOT REPLACE THE EXPERTISE OF AN EXPERIENCED CRYSTALLOGRAPHIC REFEREE.

No syntax errors found. CIF dictionary Interpreting this report

Datablock: 1

```
Bond precision:  C-C = 0.0043 A         Wavelength=0.71073

Cell:           a=8.8362(3)       b=8.8934(3)      c=14.5684(7)
                alpha=81.964(2)   beta=86.941(3)   gamma=61.769(2)
Temperature:    293 K
```

	Calculated	Reported
Volume	998.65(7)	998.65(7)
Space group	P -1	P -1
Hall group	-P 1	-P 1
Moiety formula	C12 H16 N6 O S3 Zn, 2(H2 O)	?
Sum formula	C12 H20 N6 O3 S3 Zn	C12 H20 N6 O3 S3 Zn
Mr	457.91	457.89
Dx,g cm-3	1.523	1.523
Z	2	2
Mu (mm-1)	1.566	1.566
F000	472.0	472.0
F000’	473.43	
h,k,lmax	11,11,18	11,11,18
Nref	4601	4532
Tmin,Tmax	0.855,0.984	0.855,0.984
Tmin’	0.855	

```
Correction method= # Reported T Limits: Tmin=0.855 Tmax=0.984
AbsCorr = MULTI-SCAN

Data completeness= 0.985          Theta(max)= 27.506

R(reflections)= 0.0407( 3161)     wR2(reflections)= 0.0898( 4532)

S = 1.042                  Npar= 242
```

The following ALERTS were generated. Each ALERT has the format
test-name_ALERT_alert-type_alert-level.
Click on the hyperlinks for more details of the test.

Alert level B

PLAT417_ALERT_2_B Short Inter D-H..H-D H2W ..H4W . 1.90 Ang.
x,y,z = 1_555 Check
PLAT420_ALERT_2_B D-H Without Acceptor O2W --H3W . Please Check

Alert level C

PLAT242_ALERT_2_C Low 'MainMol' Ueq as Compared to Neighbors of N4 Check
PLAT242_ALERT_2_C Low 'MainMol' Ueq as Compared to Neighbors of C11 Check
PLAT242_ALERT_2_C Low 'MainMol' Ueq as Compared to Neighbors of C12 Check
PLAT910_ALERT_3_C Missing # of FCF Reflection(s) Below Theta(Min). 9 Note
PLAT911_ALERT_3_C Missing FCF Refl Between Thmin & STh/L= 0.600 35 Report

Alert level G

PLAT002_ALERT_2_G Number of Distance or Angle Restraints on AtSite 6 Note
PLAT066_ALERT_1_G Predicted and Reported Tmin&Tmax Range Identical ? Check
PLAT172_ALERT_4_G The CIF-Embedded .res File Contains DFIX Records 1 Report
PLAT199_ALERT_1_G Reported _cell_measurement_temperature ..... (K) 293 Check
PLAT200_ALERT_1_G Reported _diffrn_ambient_temperature ..... (K) 293 Check
PLAT230_ALERT_2_G Hirshfeld Test Diff for S2 --C11 . 6.2 s.u.
PLAT230_ALERT_2_G Hirshfeld Test Diff for S3 --C12 . 5.7 s.u.
PLAT230_ALERT_2_G Hirshfeld Test Diff for N6 --C12 . 5.5 s.u.
PLAT794_ALERT_5_G Tentative Bond Valency for Zn1 (II) . 2.00 Info
PLAT860_ALERT_3_G Number of Least-Squares Restraints .... 4 Note
PLAT883_ALERT_1_G No Info/Value for _atom_sites_solution_primary . Please Do !
PLAT912_ALERT_4_G Missing # of FCF Reflections Above STh/L= 0.600 25 Note
PLAT913_ALERT_3_G Missing # of Very Strong Reflections in FCF .... 1 Note
PLAT933_ALERT_2_G Number of OMIT Records in Embedded .res File ... 18 Note
PLAT941_ALERT_3_G Average HKL Measurement Multiplicity .... 1.6 Low
PLAT978_ALERT_2_G Number C-C Bonds with Positive Residual Density. 1 Info
PLAT992_ALERT_5_G Repd & Actual _reflns_number_gt Values Differ by 1 Check

0 **ALERT level A** = Most likely a serious problem - resolve or explain
2 **ALERT level B** = A potentially serious problem, consider carefully
5 **ALERT level C** = Check. Ensure it is not caused by an omission or oversight
17 **ALERT level G** = General information/check it is not something unexpected

4 ALERT type 1 CIF construction/syntax error, inconsistent or missing data
11 ALERT type 2 Indicator that the structure model may be wrong or deficient
5 ALERT type 3 Indicator that the structure quality may be low
2 ALERT type 4 Improvement, methodology, query or suggestion
2 ALERT type 5 Informative message, check

Datablock: 2

Bond precision:	C-C = 0.0040 A		Wavelength=0.71073
Cell:	a=9.0503(4)	b=13.6692(7)	c=14.9717(8)
	alpha=90	beta=101.012(5)	gamma=90
Temperature:	293 K		

	Calculated	Reported
Volume	1818.05(16)	1818.05(16)
Space group	P 21/c	P 21/c
Hall group	-P 2ybc	-P 2ybc
Moiety formula	C12 H14 N8 S4 Zn	?
Sum formula	C12 H14 N8 S4 Zn	C12 H14 N8 S4 Zn
Mr	463.94	463.92
Dx,g cm-3	1.695	1.695
Z	4	4
Mu (mm-1)	1.824	1.824
F000	944.0	944.0
F000’	947.24	
h,k,lmax	11,17,19	11,17,19
Nref	4173	4133
Tmin,Tmax	0.896,0.913	0.852,1.000
Tmin’	0.833	

Correction method= # Reported T Limits: Tmin=0.852 Tmax=1.000
AbsCorr = MULTI-SCAN

Data completeness= 0.990 Theta(max)= 27.485

R(reflections)= 0.0356(3033) wR2(reflections)= 0.0760(4133)

S = 1.018 Npar= 230

The following ALERTS were generated. Each ALERT has the format
test-name_ALERT_alert-type_alert-level.
Click on the hyperlinks for more details of the test.

Alert level C

```
PLAT420_ALERT_2_C D-H Without Acceptor       N4       --H4A      .     Please Check
PLAT420_ALERT_2_C D-H Without Acceptor       N8       --H8A      .     Please Check
PLAT906_ALERT_3_C Large K Value in the Analysis of Variance ......      3.061 Check
```

Alert level G

```
PLAT007_ALERT_5_G Number of Unrefined Donor-H Atoms ..............          4 Report
PLAT199_ALERT_1_G Reported _cell_measurement_temperature ..... (K)        293 Check
PLAT200_ALERT_1_G Reported   _diffrn_ambient_temperature ..... (K)        293 Check
PLAT232_ALERT_2_G Hirshfeld Test Diff (M-X)  Zn1      --S2       .        7.0 s.u.
PLAT232_ALERT_2_G Hirshfeld Test Diff (M-X)  Zn1      --S4       .        5.8 s.u.
PLAT232_ALERT_2_G Hirshfeld Test Diff (M-X)  Zn1      --N6       .        5.4 s.u.
PLAT794_ALERT_5_G Tentative Bond Valency for Zn1       (II)      .       1.91 Info
PLAT883_ALERT_1_G No Info/Value for _atom_sites_solution_primary .     Please Do !
PLAT910_ALERT_3_G Missing # of FCF Reflection(s) Below Theta(Min).          4 Note
PLAT912_ALERT_4_G Missing # of FCF Reflections Above STh/L=  0.600         36 Note
PLAT941_ALERT_3_G Average HKL Measurement Multiplicity ...........        4.2 Low
PLAT978_ALERT_2_G Number C-C Bonds with Positive Residual Density.          0 Info
```

---

0 **ALERT level A** = Most likely a serious problem - resolve or explain

```
 0 ALERT level B = A potentially serious problem, consider carefully
 3 ALERT level C = Check. Ensure it is not caused by an omission or oversight
12 ALERT level G = General information/check it is not something unexpected

 3 ALERT type 1 CIF construction/syntax error, inconsistent or missing data
 6 ALERT type 2 Indicator that the structure model may be wrong or deficient
 3 ALERT type 3 Indicator that the structure quality may be low
 1 ALERT type 4 Improvement, methodology, query or suggestion
 2 ALERT type 5 Informative message, check
```

It is advisable to attempt to resolve as many as possible of the alerts in all categories. Often the minor alerts point to easily fixed oversights, errors and omissions in your CIF or refinement strategy, so attention to these fine details can be worthwhile. In order to resolve some of the more serious problems it may be necessary to carry out additional measurements or structure refinements. However, the purpose of your study may justify the reported deviations and the more serious of these should normally be commented upon in the discussion or experimental section of a paper or in the "special_details" fields of the CIF. checkCIF was carefully designed to identify outliers and unusual parameters, but every test has its limitations and alerts that are not important in a particular case may appear. Conversely, the absence of alerts does not guarantee there are no aspects of the results needing attention. It is up to the individual to critically assess their own results and, if necessary, seek expert advice.

Publication of your CIF in IUCr journals

A basic structural check has been run on your CIF. These basic checks will be run on all CIFs submitted for publication in IUCr journals (*Acta Crystallographica*, *Journal of Applied Crystallography*, *Journal of Synchrotron Radiation*); however, if you intend to submit to *Acta Crystallographica Section C* or *E* or *IUCrData*, you should make sure that full publication checks are run on the final version of your CIF prior to submission.

Publication of your CIF in other journals

Please refer to the *Notes for Authors* of the relevant journal for any special instructions relating to CIF submission.

PLATON version of 16/07/2020; check.def file version of 12/07/2020

Datablock 1 - ellipsoid plot

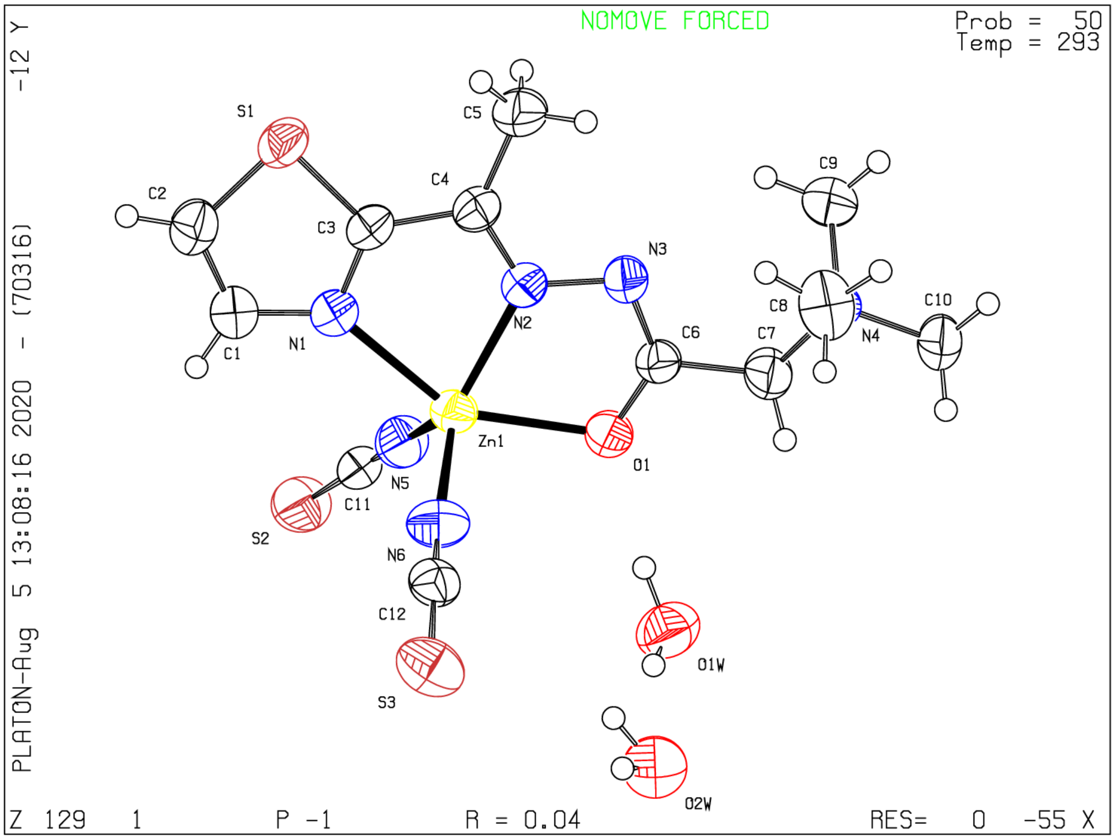

Datablock 2 - ellipsoid plot

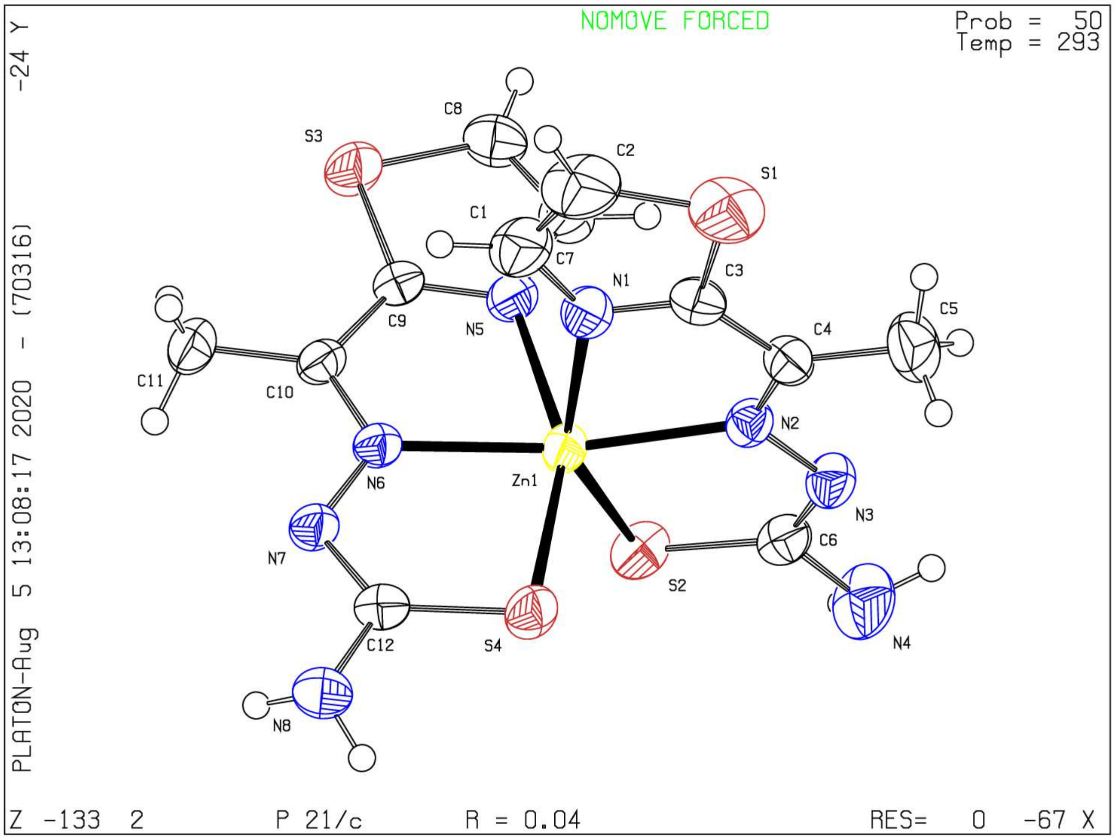

Supplement: Supplementary file 1 [file molecules-25-04043-s001.zip › molecules-915744-proofed-SM/checkcifs.pdf]
