# Supplementary material for: Synthesis, Characterization, Catalytic Activity, and DFT Calculations of Zn(II) Hydrazone Complexes
Source: Molecules. 2020 Sep 4;25(18):4043. doi: 10.3390/molecules25184043 (PMC7570652; doi:10.3390/molecules25184043)
Supplement: Supplementary file 1 [file molecules-25-04043-s001.zip › molecules-915744-proofed-SM/SI revised.docx]

Article

**Supplementary Material**

Synthesis, characterization, catalytic activity, and DFT calculations of Zn(II) hydrazone complexes

Temiloluwa T. Adejumo^1^, Nikolaos V. Tzouras^2^, Leandros P. Zorba^2^, Dušanka Radanović^3^, Andrej Pevec^4^, Sonja Grubišić^3^, Dragana Mitić^5^, Katarina K. Anđelković^1^, Georgios C. Vougioukalakis^2,^*, Božidar Čobeljić^1,^*, Iztok Turel^4,^*

^1^ Faculty of Chemistry, University of Belgrade, Studentski trg 12–16, 11000 Belgrade, Serbia; adejumo.temiloluwa@gmail.com (T.T.A.); kka@chem.bg.ac.rs (K.K.A.); bozidar@chem.bg.ac.rs (B.Č.)

^2^ Laboratory of Organic Chemistry, Department of Chemistry, National and Kapodistrian University of Athens, Panepistimiopolis, 15771 Athens, Greece; nitzouras@gmail.com (N.V.T.); leozor888@gmail (L.P.Z.); vougiouk@chem.uoa.gr (G.C.V.)

^3^ Center for Chemistry, ICTM, University of Belgrade, Njegoševa 12, P.O. Box 815, 11001 Belgrade, Serbia; radanovic@chem.bg.ac.rs (D.R.); sonja.grubisic38@gmail.com (S.G.)

^4^ Faculty of Chemistry and Chemical Technology, University of Ljubljana, Večna pot 113, 1000 Ljubljana, Slovenia; Andrej.Pevec@fkkt.uni-lj.si (A.P.); Iztok.Turel@fkkt.uni-lj.si (I.T.)

^5^ Innovation Centre of Faculty of Chemistry, University of Belgrade, Studentski trg 12–16, 11000 Belgrade, Serbia; dmitic@chem.bg.ac.rs (D.M.)

***** Correspondence: vougiouk@chem.uoa.gr; bozidar@chem.bg.ac.rs; [Iztok.Turel@fkkt.uni-lj.si](mailto:Iztok.Turel@fkkt.uni-lj.si); Tel.: +30 210 7274230 (G.C.V.); +381 69 2628978 (B.Č.); +386 1 479 8525 (I.T.)

**Table S1**. Structural parameters correlating the geometry of five-coordinate [Zn**L**X_2_] complexes (**L** = tridentate hydrazone-based ligand; X= pseudohalde, halide or DMSO).

| **Complex** | *β* (°) | *α* (°) | *τ*^1^ | *ρ*^2^ (Å) | References |
| --- | --- | --- | --- | --- | --- |
| [Zn**L^1^**(NCS)_2_]⋅2H_2_O (**1**) | 149.20(7) | 127.42(10) | 0.36 | 0.6091(3) | This work |
| [Zn**L^3^**(NCS)_2_]⋅0.5MeOH^3^ (**3**) | 147.9(2) | 128.4(3) | 0.32 | 0.6022(11) | [1] |
| [Zn**L^4^**(N_3_)_2_]^4^ (**4**) | 147.68(10) | 129.4(2) | 0.31 | 0.6165(5) | [2] |
| [Zn**L^4^**(NCO)_2_]^4^ (**5**) | 146.98(8) | 126.41(10) | 0.34 | 0.6462(4) | [2] |
| [Zn**L^4^**(N_3_)_1.65_Cl_0.35_]^4^ (**6**) | 148.40(6) | 128.68(8) | 0.33 | 0.5648(2) | [3] |
| [Zn(**L^5^**)Cl_2_]⋅0.5H_2_O^5^ (**7**) | 147.07(6) | 123.36(5) | 0.40 | 0.6453(3) | [4] |
| [Zn(**L^6^**)Cl_2_]^6^ (**8**) | 146.03(7) | 126.09(5) | 0.33 | 0.6715(2) | [5] |
| [Zn(**HL^7^**)(NCS)_2_]^7^ (**9**) | 151.16(7) | 133.96(8) | 0.29 | 0.5154(3) | [6] |
| [Zn(**HL^7^**)I_2_]^7^ (**10**) | 148.52(8) | 131.46(6) | 0.28 | 0.6628(3) | [6] |
| [Zn(**HL^7^**)Br_2_]^7^ (**11**) | 147.91(8) | 131.83(6) | 0.27 | 0.6528(3) | [6] |
| [Zn(**L^8^**)Br_2_]^8^ (**12**) | 146.06(9) | 129.81(8) | 0.27 | 0.6677(4) | [7] |
| [Zn(**L^9^**)Cl(DMSO)]^9^ (**13**) | 149.56(7) | 134.73(6) | 0.25 | 0.5025(3) | [8] |
| [Zn(**L^10^**)Cl_2_]^10^ (**14**) | 144.88(7) | 133.36(6) | 0.19 | 0.6438(3) | [9] |
| [Zn(**L^11^**)Cl_2_]^11^ (**15**) | 143.18(9) | 135.35(8) | 0.13 | 0.6503(4) | [8] |

^1^ The parameter *τ*_5_ (*τ*_5_ = (*β*−*α*)/60, where *β* and *α* are the two largest angles around the central atom) is an index of the degree of trigonality, within the structural continuum between trigonal bipyramidal and square-based pyramidal geometry.

^2^ *ρ* (Å) is the distance of metal ion from the mean basal plane of square pyramid toward the apical ligand.

^3^ **L^3^** = Condensation product of 2-acetylpyridine and trimethylammoniumacetohydrazide chloride (Girard’s T reagent).

^4^ **L^4^** = Condensation product of. 2-quinolinecarboxaldehyde and trimethylammoniumacetohydrazide chloride (Girard’s T reagent).

^5^ **L^5^** = 2-hydroxyimino-*N'*-[1-(2-pyridyl)ethylidene]propanohydrazide.

^6^ **L^6^** = (*E*)-4-(dimethylamino)-*N*′-(pyridin-2-ylmethylene)benzohydrazide.

^7^ **HL^7^** = 2-acetyl-pyridylisonicotinoylhydrazone (HAPIH).

^8^ **L^8^** = di-2-pyridyl ketone-*N*^4^-phenyl-3-semicarbazone.

^9^ **L^9^** = 2-formylpyridine-*para*-nitro-phenyl hydrazone.

^10^ **L^10^** = 2-formylpyridine isonicotinoyl hydrazone.

^11^ **L^11^** = 2-formylpyridine-*para*-chloro-phenyl hydrazone.

**Table S2.** Hydrogen-bond parameters for [Zn**L^1^**(NCS)_2_]⋅2H_2_O (**1**).

| D–H⋅⋅⋅A | D–H (Å) | H⋅⋅⋅A (Å) | D⋅⋅⋅A (Å) | D–H⋅⋅⋅A (°) | Symm.operation on A |
| --- | --- | --- | --- | --- | --- |
| O1W–H1W⋅⋅⋅O1 | 0.91(4) | 1.89(4) | 2.792(4) | 170(3) |  |
| O1W–H2W⋅⋅⋅O2W | 0.94(4) | 1.96(4) | 2.868(5) | 162(4) |  |
| O2W–H4W⋅⋅⋅S3 | 0.92(4) | 2.81(3) | 3.616(6) | 147(4) |  |
| C8–H8A⋅⋅⋅S2 | 0.96 | 2.896 | 3.737(5) | 146.8 | −1+x, 1+y, z |
| Inter layer C7–H7A⋅⋅⋅O2W | 0.97 | 2.685 | 3.575(5) | 152.7 | 1−x, 2−y, 1−z |
| C10–H10B⋅⋅⋅O1W | 0.96 | 2.686 | 3.579(5) | 155.0 | −1+x, y, z |
| Intra C5–H5B⋅⋅⋅S1 | 0.96 | 2.872 | 3.253(3) | 104.9 |  |
| Intra C9–H9B⋅⋅⋅N3 | 0.96 | 2.381 | 3.022(5) | 123.8 |  |
| Inter layer C9–H9C⋅⋅⋅O1W | 0.96 | 2.528 | 3.476(5) | 169.8 | 1−x, 1−y, 1−z |

**Table S3.** Hydrogen-bond parameters for [Zn(**L^2^**)_2_] (**2**).

| D–H⋅⋅⋅A | D–H (Å) | H⋅⋅⋅A (Å) | D⋅⋅⋅A (Å) | D–H⋅⋅⋅A (°) | Symm.operation on A |
| --- | --- | --- | --- | --- | --- |
| N4–H4B⋅⋅⋅S4 | 0.87 | 2.74 | 3.570(3) | 159.9 | 1−x, −1/2+y, 1/2−z |
| N8–H8B⋅⋅⋅S2 | 0.87 | 2.74 | 3.492(2) | 145.7 | 1−x, 2−y, −z |
| C8–H8⋅⋅⋅N3 | 0.93 | 2.73 | 3.226(3) | 114.3 | 1+x, y, z |
| Intra C5–H5B⋅⋅⋅S1 | 0.96 | 2.68 | 3.168(4) | 112.1 |  |
| Intra C11–H11B⋅⋅⋅S3 | 0.96 | 2.76 | 3.243(3) | 111.7 |  |

**Table S4.** Intermolecular π⋅⋅⋅π interaction parameters for complex **2**.

| Cg(I)^1^ | Cg(J)^1^ | Cg(I) −Cg(J)^2^ (Å) | *α*^3^ (°) | *β*^4^ (°) | *γ*^5^ (°) | Slippage^6^ (Å) | Sym. code on (J) |
| --- | --- | --- | --- | --- | --- | --- | --- |
| Cg(1) | Cg(1) | 4.0785(15) | 0.02(14) | 24.7 | 24.7 | 1.703 | 2−x, 2−y, 1−z |

^1^ Labels of aromatic rings: (1) = S1, C1−C3, N1.

^2^ Cg(I) −Cg(J) = Distance between ring centroids (Ang.).

^3^ *α* = Dihedral angle between planes (I) and (J) (Deg.).

^4^ *β* = Angle between Cg(I)−Cg(J) vector and normal to plane (I) (Deg.).

^5^ *γ* = Angle between Cg(I) −Cg(J) vector and normal to plane (J) (Deg.).

^6^ Slippage = Distance between Cg(I) and perpendicular projection of Cg(J) on ring I (Ang.).

**Table S5.** DFT calculated and experimental average values of selected bond lengths (Å) and angles (°) for [ZnL^1^(NCS)_2_] (**1**), [Zn(L^2^)_2_] (**2**) and [ZnL^3^(NCS)_2_] (**3**).

| **Compound** | **1** | | **2** | | **3** | |
| --- | --- | --- | --- | --- | --- | --- |
| Distances, Å | DFT | exp | DFT | exp | DFT | exp |
| Zn1–N6/Zn1-N6/Zn1-N6 | 1.953 | 1.955 | 2.159 | 2.149 | 1.962 | 1.965 |
| Zn1–N5/Zn1-N5/Zn1-N5 | 1.912 | 1.959 | 2.207 | 2.186 | 1.924 | 1.965 |
| Zn1–N2/Zn1-N2/Zn-N2 | 2.139 | 2.058 | 2.159 | 2.147 | 2.125 | 2.088 |
| Zn1–O1/Zn1-N1/Zn1-O1 | 1.255 | 2.178 | 1.206 | 2.318 | 1.244 | 2.206 |
| Zn1–N1/Zn1-S2/Zn1-N1 | 2.247 | 2.212 | 2.598 | 2.452 | 1.201 | 2.146 |
| O1–C6 /Zn1-S4/N3-C8 | 1.288 | 1.265 | 2.596 | 2.411 | 1.339 | 1.322 |
| Angles, ° |  |  |  |  |  |  |
| N6–Zn1–N1/N1–Zn1–N5/ N1–Zn1–N2 | 104.5 | 101.9 | 88.8 | 84.5 | 74.5 | 74.1 |
| N5–Zn1–N1/N2–Zn1–N5/ N1–Zn1–N5 | 95.2 | 97.9 | 105.2 | 103.1 | 106.2 | 100.5 |
| N5–Zn1–N2/N6–Zn1–N5/ N1–Zn1–N6 | 129.1 | 127.4 | 76.2 | 75.3 | 95.7 | 96.5 |
| N6–Zn1–O1/N2–Zn1–N1/ N1–Zn1–O1 | 91.6 | 96.9 | 76.2 | 73.6 | 145.7 | 147.9 |
| N5–Zn1–O1/N2–Zn1–S4/ N2–Zn1–O1 | 98.0 | 97.8 | 100.3 | 101.2 | 73.0 | 74.0 |

**Table S6.** E_HOMO_, E_LUMO_ and their energy gaps calculated by using TD-DFT in vacuum at different levels of theory.

|  |  | | | [Zn**L^1^**(NCS)_2_] (**1**) | | [Zn(**L^2^**)_2_] (**2**) | [Zn**L^3^**(NCS)_2_] (**3**) |
| --- | --- | --- | --- | --- | --- | --- | --- |
|  | | E_HOMO_ (eV) | | | –4.970 | –5.054 | –4.903 |
| B3LYP/6-31G | | E_LUMO_ (eV) | | | –2.803 | –2.141 | –2.438 |
|  | | *ΔE*_gap_(eV) | | | 2.167 | 2.913 | 2.465 |
|  | | | E_HOMO_ (eV) | | –5.163 | –5.245 | –5.124 |
| B3LYP/6-311G(d,p) | | | E_LUMO_ (eV) | | –2.874 | –2.306 | –2.587 |
|  | | | *ΔE*_gap_(eV) | | 2.289 | 2.939 | 2.537 |
|  | | | E_HOMO_ (eV) | | –4.417 | –4.630 | –4.371 |
| BVP86/6-311G(d,p) | | | E_LUMO_ (eV) | | –3.509 | –2.871 | –3.294 |
|  | | | *ΔE*_gap_(eV) | | 0.908 | 1.760 | 1.077 |

**Table S7.** Crystal data and structure refinement details for **1** and **2**.

|  | **1** | **2** |
| --- | --- | --- |
| formula | C_12_H_20_N_6_O_3_S_3_Zn | C_12_H_14_N_8_S_4_Zn |
| Fw (g mol^–1^) | 457.89 | 463.92 |
| crystal size (mm) | 0.10 × 0.10 × 0.01 | 0.10 × 0.05 × 0.05 |
| crystal color | colourless | yellow |
| crystal system | triclinic | monoclinic |
| space group | *P* –1 | *P* 2_1_/*c* |
| *a* (Å) | 8.8362(3) | 9.0503(4) |
| *b* (Å) | 8.8934(3) | 13.6692(7) |
| *c* (Å) | 14.5684(7) | 14.9717(8) |
| *α* (º) | 81.964(2) | 90.00 |
| *β* (º) | 86.941(3) | 101.012(5) |
| *γ* (º) | 61.769(2) | 90.00 |
| *V* (Å^3^) | 998.65(7) | 1818.05(16) |
| *Z* | 2 | 4 |
| calcd density (g cm^-3^) | 1.523 | 1.695 |
| *F*(000) | 472 | 944 |
| no. of collected reflns | 7430 | 17333 |
| no. of independent reflns | 4532 | 4133 |
| *R*_int_ | 0.0293 | 0.0450 |
| no. of reflns observed | 3161 | 3033 |
| no. parameters | 242 | 230 |
| *R*[*I*> 2σ (*I*)]*^1^* | 0.0407 | 0.0356 |
| *wR_2_*(all data)*^2^* | 0.0898 | 0.0760 |
| *Goof* , *S^3^* | 1.043 | 1.018 |
| maximum/minimum residual electron density (e Å^–3^) | +0.33/–0.29 | +0.32/–0.34 |

^1^ *R* = ∑||*F*_o_| – |*F*_c_||/∑|*F*_o_|.

*^2^ wR*_2_ = {∑[*w*(*F*_o_^2^ – *F*_c_^2^)^2^]/∑[*w*(*F*_o_^2^)^2^]}^1/2^.

*^3^ S* = {∑[*w*(*F*_o_^2^ – *F*_c_^2^)^2^]/(*n*/*p*}^1/2^ where *n* is the number of reflections and *p* is the total number of parameters refined.

| 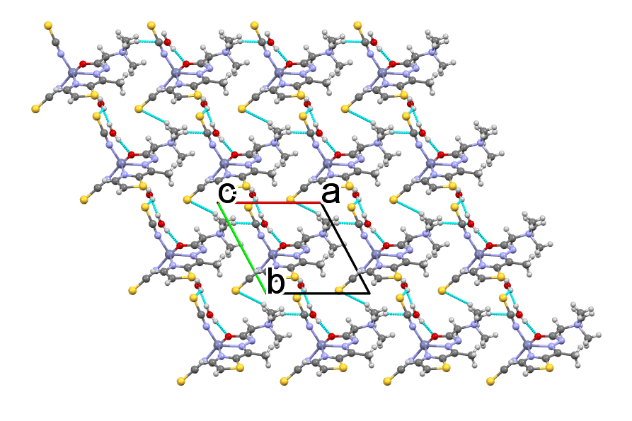  (**a**) | 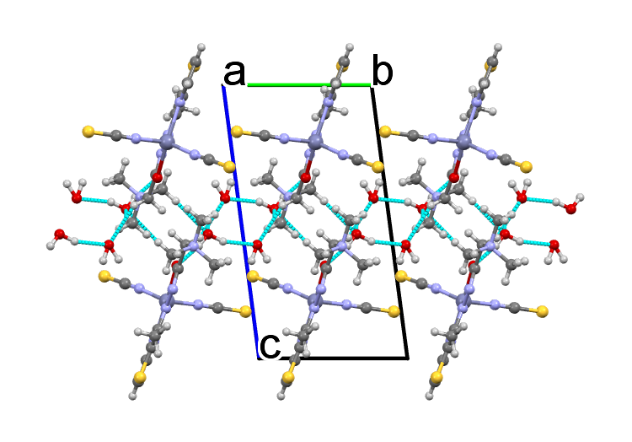  (**b**) |
| --- | --- |

**Figure s1.** (a) A view of the crystal packing of **1** showing complex molecules connected by means of OW-H⋅⋅⋅O, C_Me_-H⋅⋅⋅S and C_Me_-H⋅⋅⋅OW hydrogen bonds (dashed blue lines) into layer parallel with the (0 0 1) lattice plain. (b) A side view of the layers parallel with the (0 0 1) lattice plain related by the centers of symmetry showing the function of O1W and O2W in joining the neighboring layers.

| 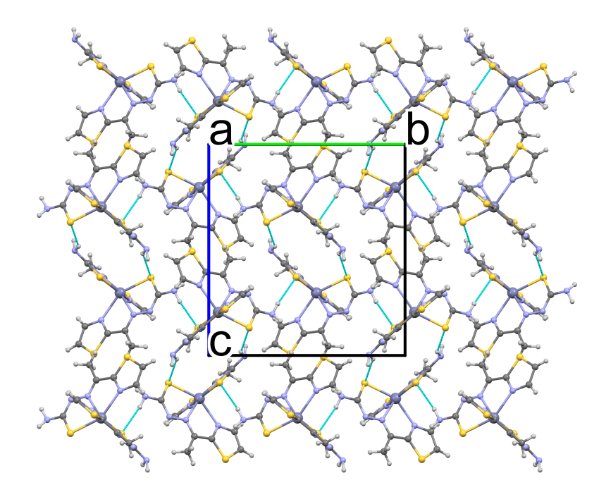  (**a**) | 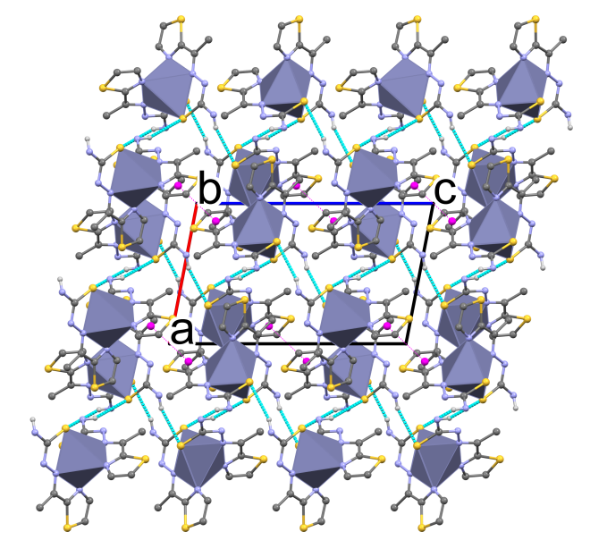  (**b**) |
| --- | --- |

**Figure s2.** (a) A view of the crystal packing of **2** showing complex molecules connected by means of N–H⋅⋅⋅S hydrogen bonds (dashed blue lines) into layer parallel with the (1 0 0) lattice plain. (b) A side view of the layers showing intermolecular *π*⋅⋅⋅*π* contacts involving 1,3-thiazole rings. Hydrogen atoms not involved in hydrogen bonding are omitted for clarity.

**^1^H and ^13^C{^1^H} NMR spectra of the synthesized propargylamines**

*1-(1-(phenylethynyl)cyclohexyl)pyrrolidine* (**4a**)

**Figure s3**. ^1^H NMR for 4a.

**Figure s4**. ^13^C NMR for 4a.

*1-(1-(phenylethynyl)cyclohexyl)piperidine* (**4b**)

**Figure s5**. ^1^H NMR for 4b.

**Figure s6**. ^13^C NMR for 4b.

*1-(1-(p-tolylethynyl)cyclohexyl)piperidine* (**4c**)


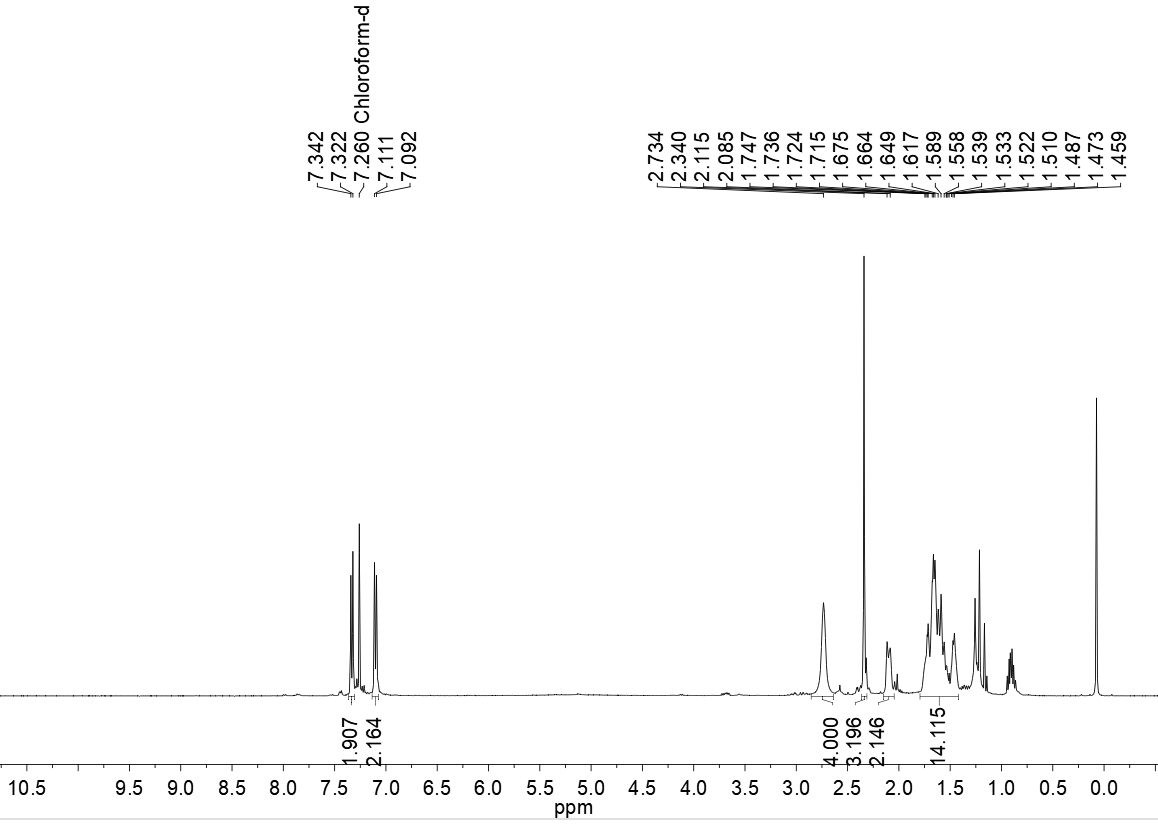


**Figure s7**. ^1^H NMR for 4c.


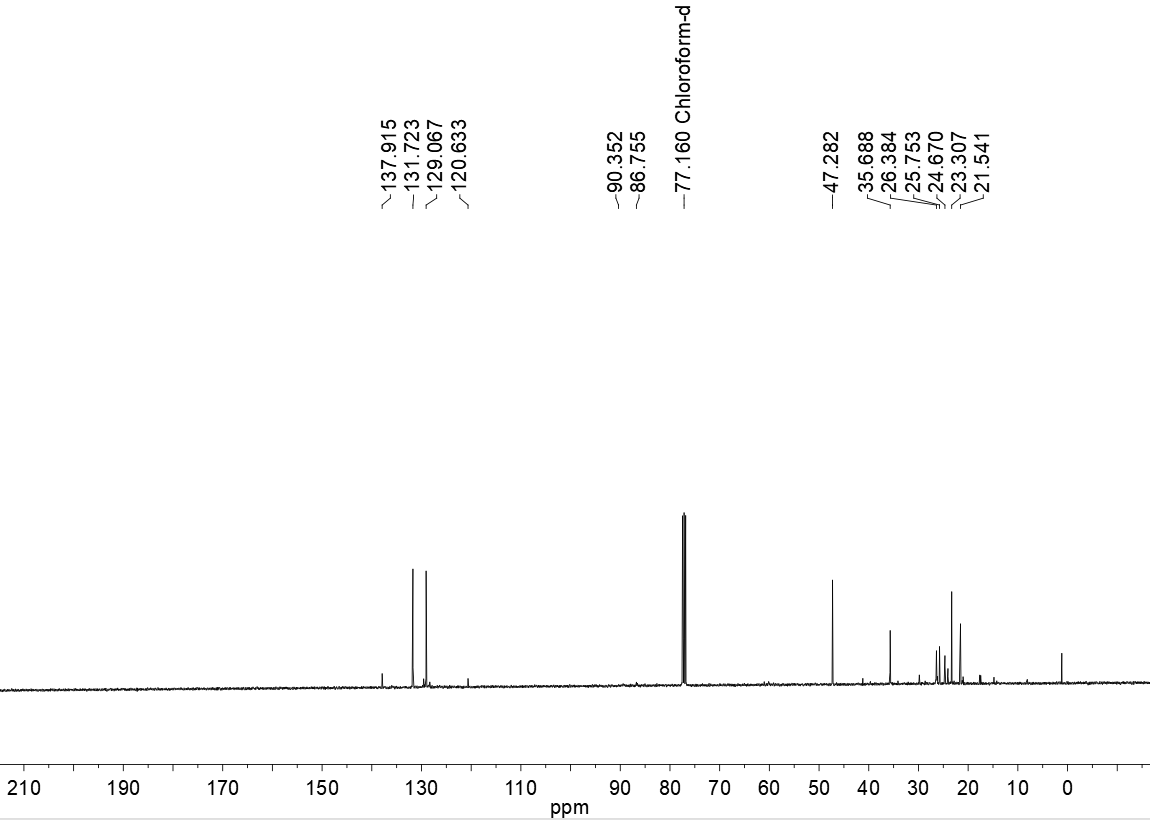


**Figure s8**. ^13^C NMR for 4c.

1*-(3-methyl-1-phenylpent-1-yn-3-yl)pyrrolidine* (**4d**)

**Figure s9**. ^1^H NMR for 4d.

**Figure s10**. ^13^C NMR for 4d.

*1-(1-(phenylethynyl)cyclohexyl)piperidine-4-carboxylate* (**4e**)

**Figure s11**. ^1^H NMR for 4e.

**Figure s12**. ^13^C NMR for 4e.

*N-octyl-1-(phenylethynyl)cyclohexanamine* (**4f**)

**Figure s13**. ^1^H NMR for 4f.

**Figure s14**. ^13^C NMR for 4f.

*1-(3-methyl-1-phenylhex-1-yn-3-yl)pyrrolidine* (**4g**)

**Figure s15**. ^1^H NMR for 4g.

**Figure s16**. ^13^C NMR for 4g.

2-methyl-4-(1-(4-phenylpiperazin-1-yl)cyclohexyl)but-3-yn-2-ol (**4h**)

**Figure s17**. ^1^H NMR for 4h.

**Figure s18**. ^13^C NMR for 4h.

*1-(1-(oct-1-yn-1-yl)cyclohexyl)piperidine* **(4i)**

**Figure s19**. ^1^H NMR for 4i.

**Figure s20**. ^13^C NMR for 4i.

*1-(1-(phenylethynyl)cyclopentyl)piperidine* (**4j**)

**Figure s21**. ^1^H NMR for 4j.

**Figure s22**. ^13^C NMR for 4j.

References

1. Čobeljić, B.; Pevec, A.; Stepanović, S.; Milenković, M.R.; Turel, I.; Gruden, M.; Radanović, D.; Anđelković, K. Structural diversity of isothiocyanato Cd(II) and Zn(II) Girard’s T hydrazone complexes in solution and solid state: effect of H-bonding on coordination number and supramolecular assembly of Cd(II) complex in solid state. *Struct. Chem.***2018**, *29*, 1797–1806.
2. Romanović, M. Č.; Čobeljić, B.; Pevec, A.; Turel, I.; Anđelković, K.; Milenković, M.; Radanović, D.; Belošević, S.; Milenković, M.R. Synthesis, crystal structures and antimicrobial activity of azido and isocyanato Zn(II)complexes with the condensation product of 2-quinolinecarboxaldehyde and Girard’s T reagent. *J. Coord. Chem*. **2017**, *70*, 2425–2435.
3. Anđelković, K.; Pevec, A.; Grubišić, S.; Turel, I.; Čobeljić, B.; Milenković, M.R.; Keškić, T.; Radanović, D. Crystal structures and DFT calculations of mixed chloride-azide zinc(II)and chloride-isocyanate cadmium(II) complexes with thecondensation product of 2-quinolinecarboxaldehyde and Girard's Treagent. *J. Mol. Struct*. **2018**, *1162*, 63–70.
4. Moroz, Y.S.; Sliva, T.Yu.; Kulon, K.; Kozłowski, H.; Fritsky, I.O. Di­chlorido{2-hy­droxy­imino-*N*′-[1-(2-pyrid­yl)ethyl­­idene]propanohydrazide-κ^3^*N*,*N*′,*O*}zinc(II) hemihydrate. *ActaCryst.* **2008**, *E64*, m353–m354.
5. Chaur, M.N. Di­chlorido{(E)-4-di­methyl­amino-*N*′-[(pyri­din-2-yl)methyl­­idene-κ*N*]benzo­hydrazide-κ*O*}zinc. *ActaCryst*. **2013**, *E69*, m27.
6. Afkhami, F.A.; Khandar, A.A.; Mahmoudi, G.; Maniukiewicz, W.; Lipkowski, J.; White, J. M.; Waterman, R.; García-Granda, S.; Zangrando, E.; Bauzái, A.; Frontera, A. Synthesis, X-ray characterization, DFT calculations and Hirshfeld surface analysis of Zn(II) and Cd(II) complexes based on isonicotinoylhydrazone ligand. *Cryst. Eng. Comm.* **2016**, *18*, 4587–4596.
7. Reena, T.A.; Seena, E.B.; Prathapachandra Kurup, M.R. Zinc(II) complexes derived from di-2-pyridyl ketone *N*^4^-phenyl-3-semicarbazone: Crystal structures and spectral studies. *Polyhedron* **2008**, *27*, 3461–3466.
8. Despaigne, A.A.R.; da Silva, J.G.; do Carmo, A.C.M.; Sives, F.; Piro, O.E.; Castellano, E.E.; Beraldo, H. Copper(II) and zinc(II) complexes with 2-formylpyridine-derived hydrazones. *Polyhedron* **2009**, *28*, 3797–3803.
9. Li, L.; Zhang, Y.Z.; Liu, E.; Yang, C.; Golen, J.A.; Rheingold, A.L.; Zhang, G. Synthesis and structural characterization of zinc(II) and cobalt(II) complexes based on multidentatehydrazone ligands. *J. Mol. Struct*. **2016**, *1110*, 180–184.
